# Supplementary material for: The VNTR 48 bp Polymorphism in the DRD4 Gene Is Associated with Higher Tobacco Smoking in Male Mexican Mestizo Smokers with and without COPD
Source: Diagnostics (Basel). 2019 Dec 30;10(1):16. doi: 10.3390/diagnostics10010016 (PMC7168062; doi:10.3390/diagnostics10010016)
Supplement: Supplementary file 1 [file diagnostics-10-00016-s001.pdf]

Supplementary tables

Supplementary Table 1. Allele and genetics models in comparison to COPD and HS.

Allele/ Codominant	COPD (n=164)		HS (n=164)		p-value
	n	AF/GF%	n	AF/GF%	
S	213	64.93	222	67.68	0.508
L	115	35.06	106	32.31	
SS	71	43.3	75	45.73	0.463
SL	71	43.3	72	43.9	
LL	22	13.4	17	10.36	
Dominant	n	F %	n	F %	
SS	71	43.3	75	45.7	0.738
SS+SL	93	56.7	89	54.3	
Recessive	n	F %	n	F %	
SS+SL	142	86.6	147	89.6	0.495
LL	22	13.4	17	10.4	

Abbreviations: COPD, Chronic obstructive pulmonary disease; HS, heavy smokers; AF, allele frequencies; GF, genotype frequencies; F, frequencies. p-value by Yate's correction.

Supplementary Table 2. Allele and genetics models in comparison to COPD and LS.

**Allele/ Codominant**	**COPD** (n=164)		**LS** (n=164)		**p-value**
	n	AF/GF%	n	AF/GF%	
S	213	64.93	228	69.51	0.244
L	115	35.06	100	30.48	
SS	71	43.3	84	51.21	0.233
SL	71	43.3	60	36.58	
LL	22	13.4	20	12.19	
**Dominant**	**n**	**F %**	**n**	**F %**	
SS	71	43.3	84	51.2	0.184
SS+SL	93	56.7	80	48.8	
**Recessive**	n	F %	n	F %	
SS+SL	142	86.6	144	87.8	0.868
LL	22	13.4	20	12.2	

Abbreviations: COPD, Chronic obstructive pulmonary disease; LS, light smokers; AF, allele frequencies; GF, genotype frequencies; F, frequencies. p-value by Yate's correction.

Supplementary Table 3. Allele and genetics models in comparison to HS and LS.

**Allele/ Codominant**	**HS** (n=164)		**LS** (n=164)		**p-value**
	n	AF/GF%	n	AF/GF%	
S	222	67.68	228	69.51	0.674
L	106	32.31	100	30.48	
SS	75	45.73	84	51.21	0.625
SL	72	43.9	60	36.58	
LL	17	10.36	20	12.19	

Dominant	n	F %	n	F %	
SS	75	45.7	84	51.2	0.376
SS+SL	89	54.3	80	48.8	
**Recessive**	n	F %	n	F %	
SS+SL	147	89.6	144	87.8	0.727
LL	17	10.4	20	12.2	

Abbreviations: HS, heavy smokers; LS, light smokers; AF, allele frequencies; GF, genotype frequencies; F, frequencies. p-value by Yate's correction.

Supplementary Table 4. Comparison by age of onset in all populations of the study, in women and men.

Allele/ Codominant	**<18 years** (n=297)		**≥18 years** (n=195)		**p-value**
	n	AF/GF%	n	AF/GF%	
S	386	64.9	268	68.7	0.252
L	208	35.1	122	31.3	
SS	129	43.43	94	48.2	0.496
SL	128	43.10	80	41	
LL	40	13.47	21	10.7	
Dominant	n	F %	n	F %	
SS	129	43.43	94	48.21	0.343
SL+LL	168	56.57	101	51.79	
Women	**<18 years** (n=90)		**≥18 years** (n=90)		
Allele/ Codominant	n	AF/GF%	n	AF/GF%	
S	113	62.7	125	69.5	0.220
L	67	37.2	55	30.5	
SS	37	41.1	43	47.7	0.189
SL	39	43.3	39	43.3	
LL	14	15.5	8	8.9	
Dominant	n	F %	n	F %	
SS	37	41.11	43	47.78	0.453
SL+LL	53	58.89	47	52.22	
Men	**<18 years** (n=208)		**≥18 years** (n=104)		
Allele/ Codominant	n	AF/GF%	n	AF/GF%	
S	270	64.9	150	72.1	0.085
L	146	35.1	58	27.9	
SS	91	43.8	56	53.8	0.082
SL	88	42.3	38	36.5	
LL	29	13.9	10	9.6	
Dominant	n	F %	n	F %	
SS	91	43.75	56	53.85	0.117
SL+LL	117	56.25	48	46.15	

Abbreviations: AF, allele frequencies; GF, genotype frequencies; F, frequencies. p-value by Yate's correction.

Supplementary Table 5. Comparison by sex in LS, HS and COPD groups.

LS	Men (n=80)		Women (n=84)		p-value
Allele/ Codominant	n	AF/GF%	n	AF/GF%	
S	117	73.1	108	64.3	0.108
L	43	26.9	60	35.7	
SS	47	58.8	35	41.7	0.106
SL	23	28.7	38	45.3	
LL	10	12.5	11	13	
**Dominant**	n	F %	n	F %	
SS	47	58.75	35	41.67	0.042
SL+LL	33	41.25	49	58.33	
HS	**Men (n=93)**		**Women (n=71)**		
Allele/ Codominant	n	AF/GF%	n	AF/GF%	
S	123	66.1	94	66.2	0.165
L	63	33.9	48	33.8	
SS	40	43	32	45	0.989
SL	43	46.2	30	42.2	
LL	10	10.7	9	12.7	
**Dominant**	n	F %	n	F %	
SS	40	43.01	32	45.07	0.916
SL+LL	53	56.99	39	54.93	
COPD	**Men (n=130)**		**Women (n=34)**		
Allele/ Codominant	n	AF/GF%	n	AF/GF%	
S	165	63.5	47	69.1	0.467
L	95	36.5	21	30.9	
SS	53	40.7	16	47.1	0.389
SL	59	45.4	15	44.1	
LL	18	13.8	3	8.8	

Dominant	n	F %	n	F %	
SS	53	40.77	16	47.06	0.640
SL+LL	77	59.23	18	52.94	

Abbreviations: LS, light smokers; HS, heavy smokers; COPD, Chronic obstructive pulmonary disease; AF, allele frequencies; GF, genotype frequencies; F, frequencies. p-value by Yate's correction.

Supplementary Table 6. Comparison in women included in this study.

Codominant	HS (n=71)		LS (n=84)		p-value
	n	GF%	n	GF%	
SS	32	45	35	41.7	0.730
SL	30	42.2	38	45.3	
LL	9	12.7	11	13	
**Dominant**	n	F %	n	F %	
SS	32	45	35	41.7	0.792
SL+LL	39	54.9	49	58.3	
Codominant	**COPD (n=34)**		**LS (n=84)**		
	n	GF%	n	GF%	
SS	16	47.1	35	41.7	0.481
SL	15	44.1	38	45.3	
LL	3	8.8	11	13	
**Dominant**	n	F %	n	F %	
SS	16	47	35	41.7	0.741
SL+LL	18	52.9	49	58.3	
Codominant	**COPD (n=34)**		**HS (n=71)**		
	n	GF%	n	GF%	
SS	16	47.1	32	45	0.679
SL	15	44.1	30	42.2	
LL	3	8.8	9	12.7	
**Dominant**	n	F %	n	F %	
SS	16	47	32	45	0.985
SL+LL	18	52.9	39	54.9	

Abbreviations: HS, heavy smokers; LS, light smokers; COPD, Chronic obstructive pulmonary disease; GF, genotype frequencies; F, frequencies. p-value by Yate's correction.

Supplementary Table 7. Comparison only in men included in this study.

Codominant	COPD (n=130)		HS (n=93)		p-value
	n	GF%	n	GF%	
SS	53	40.7	40	43	0.562
SL	59	45.4	43	46.2	
LL	18	13.8	10	10.7	
**Dominant**	n	F %	n	F %	
SS	53	40.7	40	43	0.843
SL+LL	77	59.2	53	57	

Abbreviations: COPD, Chronic obstructive pulmonary disease; HS, heavy smokers; GF, genotype frequencies; F, frequencies. p-value by Yate's correction.
